# Depth-based local registration refinement for augmented reality in pituitary surgery

**DOI:** 10.1007/s11548-026-03705-0

**Published:** 2026-06-05

**Authors:** Aure Enkaoua, Manuel Villa, João Ramalhinho, Mobarak I. Hoque, Hani J. Marcus, Matthew J. Clarkson

**Affiliations:** 1 UCL Hawkes Institute, Department of Medical Physics and Biomedical Engineering, University College London, London, UK; 2 National Hospital for Neurology and Neurosurgery, London, UK; 3 CEIMM, Universidad Politécnica de Madrid, Madrid, 28031 Spain; 4 Division of Informatics, Imaging and Data Science, UoM, Manchester, UK

**Keywords:** Augmented Reality, Transsphenoidal Pituitary Surgery, Minimally Invasive Surgery, Registration, Endoscopic Surgery

## Abstract

**Purpose:**

Accurate registration between pre-operative anatomical models and intra-operative endoscopic views is essential for reliable Augmented reality (AR) guidance in pituitary surgery. While coarse alignment can be achieved using external facial reconstruction, it does not account for relative motion between the face and internal surgical targets. Precise alignment at the sella region further remains challenging due to the lack of direct depth information in monocular endoscopy.

**Methods:**

To identify an appropriate similarity metric for registration refinement, we conducted a grid search comparing monocular depth predictions from endoscopic images with depth rendered from a pre-operative 3D model. Normalised cross-correlation (NCC) demonstrated the most robust performance and was selected as the optimisation objective. A continuous optimisation framework was then implemented to iteratively refine translational pose parameters by maximising NCC, with optimisation applied periodically and propagated across frames for computational efficiency.

**Results:**

Depth-based optimisation improved alignment at the surgical target, increasing DICE from 0.77 to 0.81, IoU from 0.63 to 0.68, and reducing centroid localisation error by 36%.

**Conclusion:**

Overall, the proposed depth-based refinement improves target alignment in endoscopic AR guidance and demonstrates the potential of monocular depth cues for intra-operative registration correction.

## Introduction

Augmented reality (AR) has been explored in pituitary surgery to overlay pre-operative anatomical information onto live endoscopic video, using infrared reflective markers [3], visual markers [8] and markerless approaches [6, 7].

A key requirement for effective AR guidance is accurate registration between pre-operative anatomical models and intra-operative endoscopic views. While coarse registration can be achieved using strategies such as manual alignment, marker-based registration, external surface matching, or anatomical landmarks, achieving accurate alignment at the anatomical region of clinical interest remains challenging. This is because coarse alignment based on external anatomy does not account for relative motion between the face and internal surgical targets, and therefore misalignment can persist or increase as the endoscope approaches the sella region.

Accurate registration refinement at the region of interest (the sella turcica in pituitary surgery) requires both modalities to be represented in a common coordinate space. Although pre-operative scans provide 3D anatomical information, monocular endoscopic images lack depth, making direct 3D alignment difficult. Several methods have been proposed to infer depth [10] or reconstruct 3D structures from monocular endoscopy, but these approaches often suffer from scale ambiguity relative to the pre-operative scan [5].

In this work, we extend our previous marker-based AR system with automatic registration [1] by introducing a local depth-based registration refinement strategy. The proposed method uses monocular depth prediction from endoscopic images and depth rendered from a coarsley registered pre-operative 3D model to improve alignment directly at the surgical target.

## Methods

**Fig. 1:**
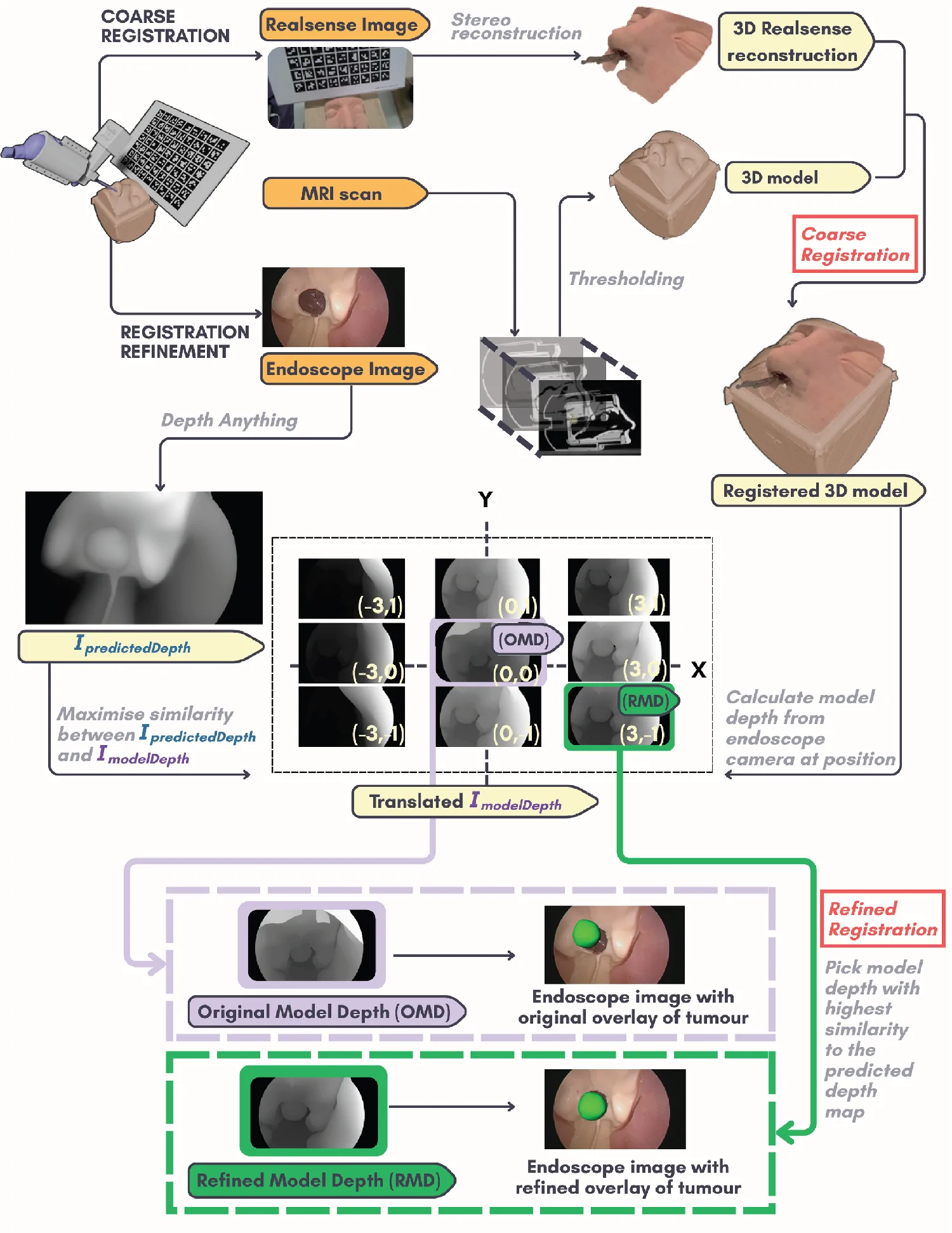
System overview of the proposed AR-guided registration refinement framework. Orange boxes indicate the data inputs: a pre-operative MRI scan, an external stereo RealSense camera, and a monocular endoscopic camera. The coarse registration step aligns the reconstructed facial surface to the 3D face model derived from the MRI scan. The refinement step improves alignment at the surgical target: depth is predicted from the endoscopic image using depth anything, while a synthetic depth image is rendered from the registered 3D model at the current camera position. To find a better alignment, a local search, either an exhaustive grid search or continuous optimisation, identifies the translated model pose whose rendered depth best matches the predicted depth. The Original Model Depth (OMD, purple) shows the starting position after coarse registration, and the Refined Model Depth (RMD, green) represents the optimal pose found, which is used to update the AR tumour overlay shown to the surgeon

Our AR system for pituitary surgery [1] uses an external stereo camera mounted on the endoscope to reconstruct the facial surface. This reconstruction is used to perform automatic coarse registration with a pre-operative face model derived from the scan. However, this step does not account for relative motion between the face and internal surgical targets, leading to misalignment as the endoscope approaches the sella region. The full pipeline of the proposed system is shown in Fig. 1.

To refine the registration locally, a synthetic depth image is rendered from the registered 3D anatomical model using z-buffer rendering and known camera intrinsics, denoted as $I_{modelDepth}$. A depth map is then inferred from the monocular endoscopic image using the Depth Anything framework [9], denoted as $I_{predictedDepth}$. The goal of the refinement step is to maximise the similarity between these two depth images.

### Similarity metric evaluation via grid search

To select an appropriate similarity metric for depth-based refinement, we first perform an exhaustive grid search over small pose perturbations. Starting from the initial coarse registration, the model pose is systematically translated along the two in-plane directions (X and Y), with offsets sampled between -3 and 3 mm at 1 mm intervals. For each candidate pose, similarity between $I_{modelDepth}$ and $I_{predictedDepth}$ is evaluated using several metrics: Mean Squared Error (MSE), Normalised Cross-Correlation (NCC), Structural Similarity Index (SSIM), and feature-based cosine similarity derived from deep network embeddings (VGG and DINO). This analysis allows us to compare how different similarity measures behave for depth-based refinement at the surgical target and to identify the most robust metric for optimisation.

### Segmentation-based evaluation

To assess refinement accuracy at the surgical target, we perform a segmentation-based evaluation of the tumour region. Tumours are segmented independently in three images: (1) the original endoscopic image, (2) the AR overlay after coarse registration, and (3) the AR overlay after refinement. Binary tumour masks are generated using Grounded SAM 2 [2, 4].

Registration accuracy is quantified using the DICE coefficient, Intersection-over-Union (IoU) and centroid distance between segmentations. These metrics provide an estimate of target registration error (TRE) at the region of interest.

### Continuous registration refinement via optimisation

During runtime, registration refinement is performed using an optimisation-based approach with the similarity metric selected during grid search. Starting from the coarse registration, the pose is iteratively refined by maximising NCC between $I_{modelDepth}$ and $I_{predictedDepth}$.

Unlike the grid search, which considers only two in-plane translations, the optimisation framework refines all three translational degrees of freedom (DOF), including motion along the camera viewing direction. This enables correction of depth misalignment between the endoscope and the anatomical model that cannot be resolved through shifts along the image plane alone. To balance computational cost and temporal stability, optimisation is performed every 10 frames, with the resulting transformation propagated to intermediate frames.

## Experiment and results

We evaluate the proposed framework in two stages. First, an exhaustive local grid search is used to compare similarity metrics and identify the most robust objective function for depth-based refinement. The selected metric is then incorporated into a continuous optimisation framework, whose performance is evaluated across multiple recordings in a realistic runtime setting.

### Similarity metric comparison via grid search

**Fig. 2:**
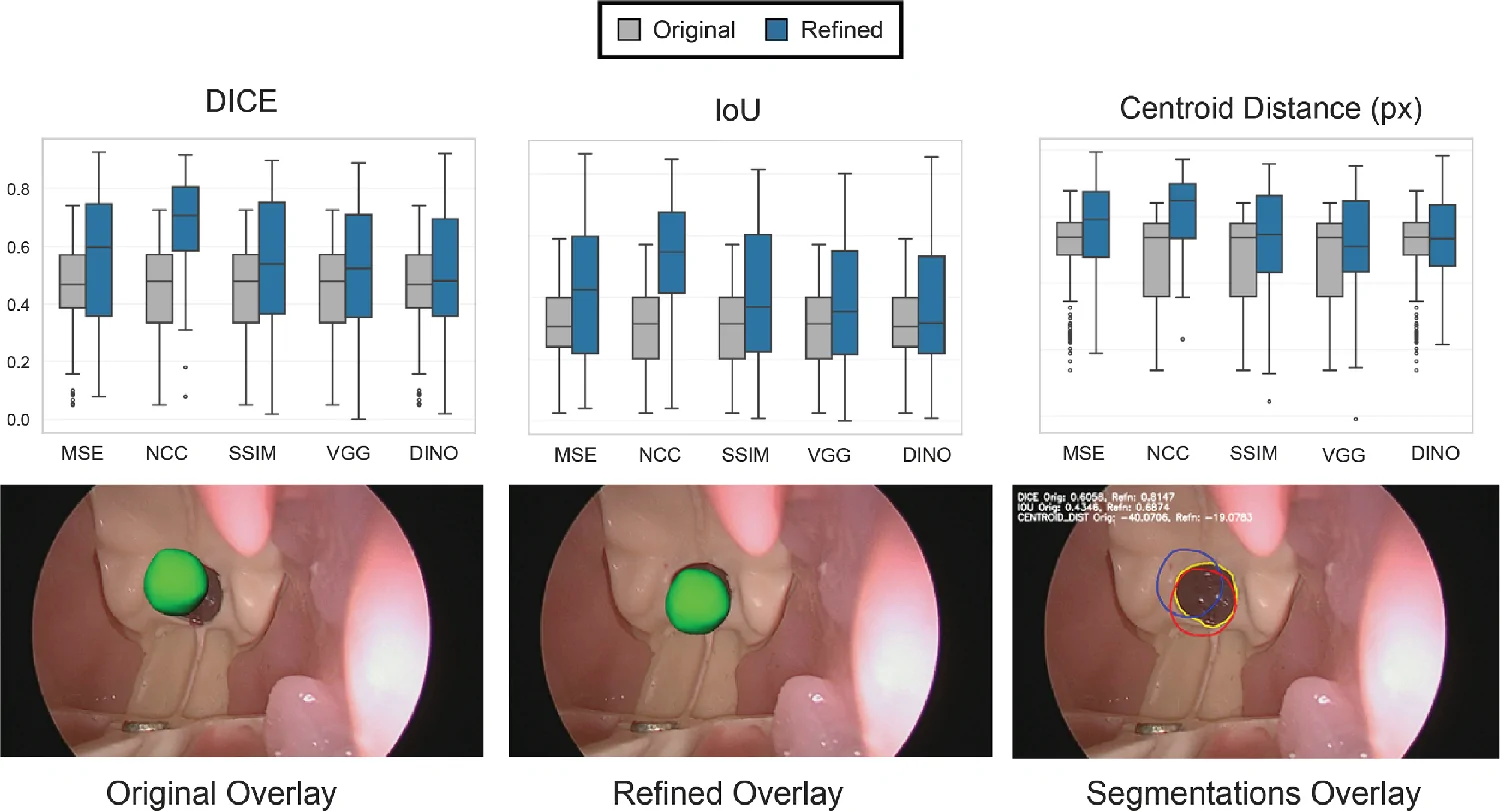
Quantitative and qualitative evaluation of depth-based registration refinement grid search metric comparison. Top row: box plots showing DICE coefficient, Intersection-over-Union (IoU), and centroid distance for the original overlay (light grey) and refined overlay (blue), aggregated across all recordings. Centroid distance is negated so that higher values indicate better alignment across all metrics. Bottom row: qualitative examples showing original AR overlay (left), refined AR overlay (middle), and overlay of tumour contours (right), where ground-truth tumour segmentation is outlined in yellow, the original overlay in blue, and the refined overlay in red

Figure 2 summarises both the quantitative and qualitative impact of the refinement across all evaluated frames. Across all segmentation metrics, refinement using NCC consistently provides the largest improvement. The median DICE score increases from 0.48 to 0.71, while the median IoU rises from 0.32 to 0.55, and the median centroid distance is reduced by approximately 28 pixels. Other measures, including MSE, SSIM, VGG, and DINO, show smaller and less consistent gains. Based on these findings, NCC was selected as the objective function for continuous registration refinement.

### Continuous refinement via optimisation

To evaluate performance in a continuous setting, we apply the NCC-based optimisation framework to 10 independent endoscopic recordings comprising a total of 354 frames. Optimisation is performed every 10 frames, and the resulting transformation is propagated to intermediate frames.

Table 1 summarises the quantitative comparison between the original coarse registration and the refined alignment, reported as median values and interquartile ranges (IQR) across all evaluated frames.

**Table 1:** Registration accuracy before and after NCC-based optimisation across 354 frames from 10 recordings.

Metric	Original	Optimised
DICE	0.77 [0.06]	0.81 [0.08]
IoU	0.63 [0.07]	0.68 [0.11]
Centroid Distance (pixels)	61.2 [13.8]	39.1 [25.7]

Values reported as median [IQR]

Optimisation consistently improves alignment at the surgical target. Relative to the coarse registration, segmentation overlap increases by approximately 5%, while tumour localisation error is reduced by approximately 36%. These results demonstrate that continuous depth-based refinement provides consistent improvements in both overlap accuracy and spatial localisation.

## Discussion and conclusion

We present a depth-based local registration refinement framework that combines monocular depth prediction from endoscopic images with depth rendered from a pre-operative 3D model. By directly comparing these depth images at the surgical target, the method improves AR overlay alignment where it is most clinically relevant. This is particularly important in pituitary surgery, where small misalignments at the sella can directly affect clinical outcomes.

Among all similarity metrics evaluated, NCC demonstrated the most consistent behaviour during grid search, likely due to its invariance to linear intensity scaling and bias, which is an issue when comparing predicted and rendered depth maps. A continuous optimisation strategy was therefore adopted, refining the pose in three translational DOF by maximising NCC between the predicted and rendered depth. Consistent improvements across DICE, IoU, and centroid distance suggest that monocular depth cues provide a reliable signal for local registration correction at the surgical target.

The proposed framework represents, to our knowledge, the first AR registration system that combines tracked automatic coarse registration with vision-based local refinement at the surgical target. A key advantage is that refinement is performed directly at the region of clinical interest, which coarse registration methods based on facial alignment cannot reliably achieve. The system combines the stability of tracked global registration with the flexibility of vision-based local correction, and runs fully automatically without manual intervention during surgery.

This study has several limitations. All recordings were collected on a surgical phantom, which does not fully capture the complexity of real surgery, including tissue movement, fluid presence, occlusions, and illumination changes. In addition, the current method refines each frame independently using only translational corrections, which limits its ability to model more complex motion and temporal consistency. Future work will extend the optimisation to include rotational parameters and exploit temporal coherence across frames, for example through joint multi-frame optimisation. Further validation on clinical data will also be required to assess robustness in real surgical conditions.
